# Supplementary figures and images for: Effects of larval rearing substrates on some life-table parameters of Lutzomyia longipalpis sand flies
Source: PLoS Negl Trop Dis. 2021 Jan 21;15(1):e0009034. doi: 10.1371/journal.pntd.0009034 (PMC7870073; doi:10.1371/journal.pntd.0009034)

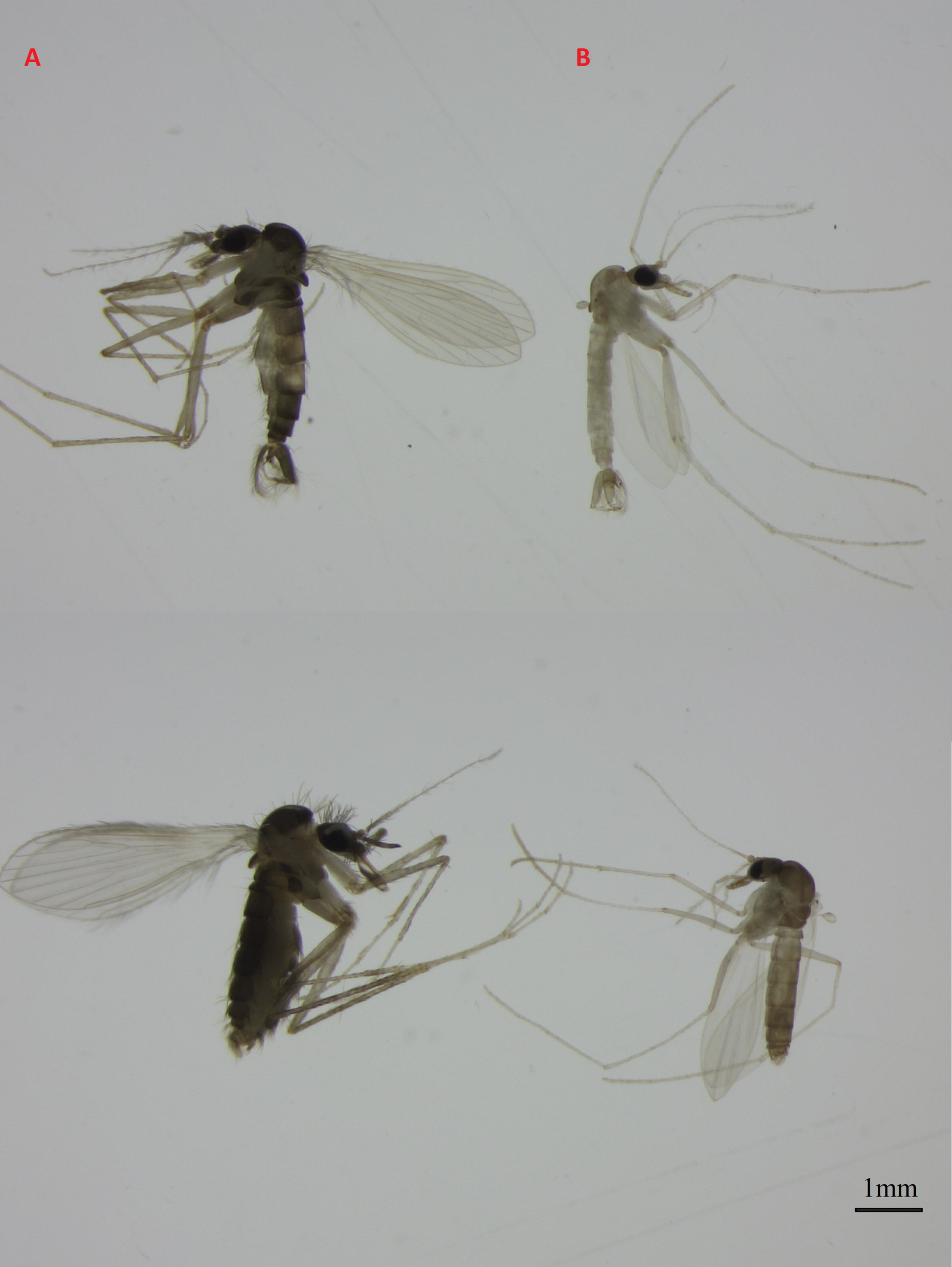

Supplement: S1 Fig — Males and females reared on substrates from A) chicken coops (left), and B) composting Cashew tree leaf litter (right). (TIF) [file pntd.0009034.s001.tif]

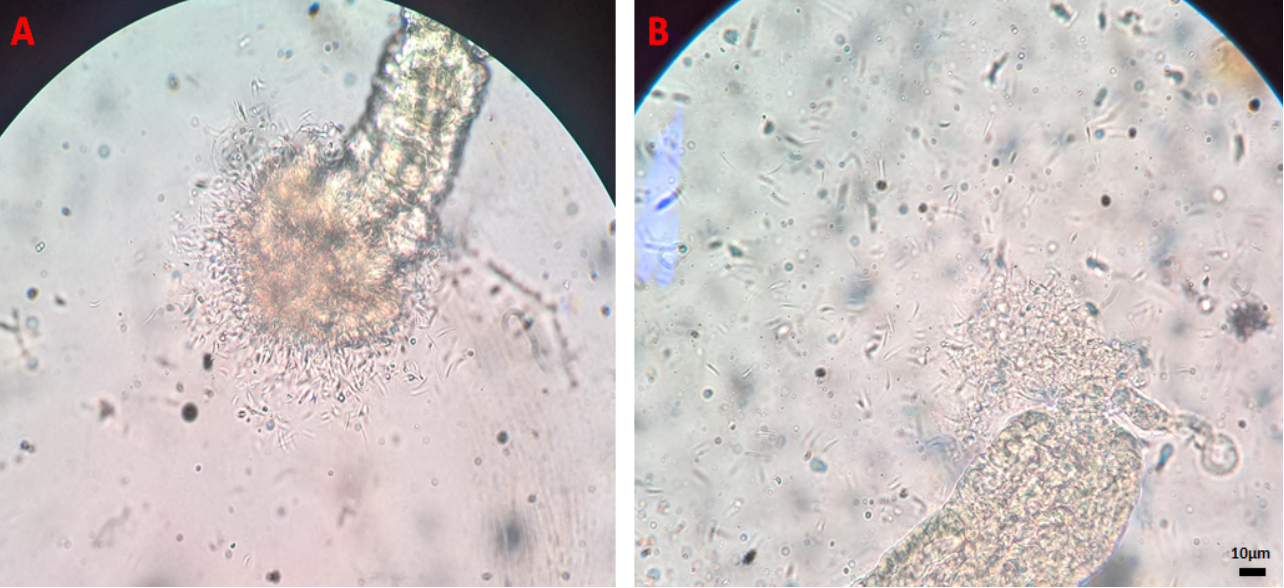

Supplement: S2 Fig — A) dissection of female reared in chicken coops, and B) dissection of female reared with colony food. (TIF) [file pntd.0009034.s002.tif]
