# Supplementary material for: Effects of larval rearing substrates on some life-table parameters of Lutzomyia longipalpis sand flies
Source: PLoS Negl Trop Dis. 2021 Jan 21;15(1):e0009034. doi: 10.1371/journal.pntd.0009034 (PMC7870073; doi:10.1371/journal.pntd.0009034)
Supplement: Attachment — Submitted filename: Referee 2 report.docx [file pntd.0009034.s004.docx]

Dear Editor,

I would like to thank you very much for choosing my expertise to review this highly interesting and unique piece of work.

Briefly, the breeding sites of sand fly vectors of Leishmaniasis remain an unsolved mystery, preventing public health authorities from launching targeted measures to prevent and control this disfiguring and lethal disease, in some cases.

The authors have cleverly and originally examined several different substrates of putative breeding sites where adult sand flies can be collected, from an endemic area of visceral leishmaniasis in Camacari, Bahia, Brazil and have studied them in depth in light of their: (1) chemical and nutritional compositions, (2) oviposition capabilities, (3) larval development, (4) larval microbiome structure and (5) susceptibility to attach, develop, and sustain Leishmania infantum parasites in sand flies reared on them.

After an extensive review of the manuscript number PNTD-D-20-01426 **- Effects of larval rearing substrates on some life-table parameters of *Lutzomyia longipalpis* sand flies,** I would like to recommend it for publication, once the following comments and suggestions have been addressed by the authors and corrections, or justifications, have fully satisfied this reviewer.

However; before moving forward with my comments and suggestions, I would like to advise the authors to give the paper to a native English speaker for assessment of proper English usage. Here in the US, there are several English teachers, native speakers, who could do it for a small fee. I was able to catch a few mistakes here and there throughout the text.

Line 50: Ecological niches versus habitats. What was the intention behind of the sentence “Insects were reared on substrates from different ecological niches”….? Was it a way of emphasizing the position of *Lutzomyia longipalpis* within an ecosystem, describing both the range of conditions necessary for insect persistence and its ecological role or merely to inform the putative different substrates where the flies reside?

Line 66: Remove: “that had fed on”

Add: “who feed on infected hosts”….

Line 67-70: It would worth mentioning the mucocutaneous form of the disease; because of its uniqueness and close correlation with some Leishmania species.

Line 76: I praise the authors for explicitly recognizing *Lutzomyia longipalpis* as a complex of species, well overdue by the sand fly community!

Line 76: Remove: “where sand flies were feeding on…” Add: where sand flies used to feed exclusively on wild animals and plants; however, with the …..

Line 82: comprises of residual spraying…..

Line 86: Add: the word humus after “decaying organic matter or humus.

Line 95: Suggestion: Although, the term “microbiota” of sand flies was employed correctly, I would review the entire manuscript for consistency; always having in mind the differences between “microbiome” and “microbiota”.

Line 106 (Study Sites): I would include a brief description of the flora of Jaua and Jacuipe, since substrates from different tree species collected in both areas were analyzed in this study. Is one of these tree substrates unique to one of the areas? Is the “coastal strip”, a remaining patch of the extensively deforested Atlantic Forest? Please, specify it.

Line 114 (Insects): Since the foundation of all experiments relied heavily on colonized flies, with either eggs or adults, it is imperative to specify the following:

(1) How many generations the *L. longipalpis* colony, used for all experiments, has been kept in the lab? When was it stablished?
(2) How big is the colony?

(3) What is the yearly production of the colony used for the experiments? Is it mass reared?

(4) If it is a closed and old colony, how inbreeding and selective pressures by daily rearing methods could impact the outcome of the experiments? If the experiments were done with F1 progeny straight from the field, I assume the authors have access to field collected flies, would it have changed the outcome of the experiments? Please, add some comments on this regard to the discussion section too.

(5) What was the brand of rabbit feed used for maintaining the colony? What was its composition (examples: reach in what nutrients?, specially made or commercially available formula?).

(6) What was the source of rabbit feces? Were the rabbits maintained at Fiocruz or bought from outside vendors? Did they receive any antibiotic treatment that might impair the development and microbiome change of larval stages? What was the diet given to the rabbits? Were they kept on an organic diet?

I would add that larval food was made according to Modi and Tesh, 1983* protocol with a few modifications.

*References provided at the end of this review.

Lines 127-132 (Substrates): I am really puzzled by this method. How did the authors manage to control fungi growth in larval jars treated with different substrates and applied directly from the field, since the traditional sand fly food preparation method, originally proposed by Young et.al (1981) and latter on simplified by Modi and Tesh in 1983, requires larval food to decompose or “age” for a period of at least 4-6 weeks in a highly humid environment in order for fungi to grow freely, preventing their appearance and trapping of larvae latter on during the sand fly rearing?

“Material was dried, crushed, sieved and stored”…

What was the method used for drying the different substrates? Was it submitted to high temperatures or just air dried? Please specify it, because high temperatures tend to deplete nutrients altering larvae microbiome, this is why larval food shouldn’t be autoclaved, particularly when using flies for artificial infection latter on.

Lines 137-149 (Oviposition Assays): Comment: According to my experience, it would have made a huge difference, if authors had prime impregnated the pots overnight with “FRASS” solution of each individual substrate prior to adding the flies; instead of using the substrates directly, which in my view were highly susceptible to developing mold in the high moisture and temperature conditions.

Please refer to the work of Wasserberg and Rowton (2011)*. This is done regularly to rapidly increase colony flies, regardless of species, with great improvement in number of eggs oviposited by each female fly!!!

Line 139: Please specify the pot or vial used (brand, catalog number, size, etc.). Were they Falcon tubes? Self-standing tubes? 3-dram vials?

Line 140: Not all plasters are created equal! Please specify the brand used, if dental or plaster for dry wall construction (still releasing chemicals that could impact egg laying).

Also, old pots (pots that have passed through the colony several times) tend to be more productive in terms of egg laying, since they retain better moisture and “odors” released by female flies during ovipositing and humus impregnating, refer to Wasserberg and Rowton work, than freshly made ones. Did the authors consider this critical piece of information when designing the experiments for this study?

Line 144: How did the authors determine the optimal number of females to be confined in each pot? Did they take into account that aggregation plays a major role in sand fly ovipositing? Why male flies were not added to each pot? Some laboratory observations suggest that male flies help during ovipositing and blood meal acquiring of female flies. We have observed a significant improvement in sand fly artificial feeding when males (5-10% of total) are added to containers with females. Did the authors take into account the size of the pots and the fact that *L. longipalpis* oviposits better when confined in tiny spaces? How was the density of flies per area (cm^2^) determined? Empirically?

Line 145: Under standard laboratory conditions of 65%RH - 27^0^C, used for general rearing, *L. longipalpis* flies require an average of 7 to 10 days to fully oviposit their eggs. This can be accelerated or retarded by changing temperature, humidity and photoperiod conditions. Questions:

(1) Why did the authors decide to interrupt the egg laying after 72 hours? What was the reasoning behind it? Did the adult flies simply die after 72 hours or was it cut short because mold from raw substrates started building up, trapping the flies? This is why I continue to insist that FRASS impregnation would have helped a lot!
(2) If they had left the flies for a longer period, would they have oviposited more eggs per substrate?
(3) Did the authors consider the possibility of autogeny, already described in long term colonized flies, when designing the experiments?

Line 148: I am highly puzzled by this method too! How were the small microscopic sand fly eggs of just three females scooped out from pots and counted, while still mixed with the different substrates? This alone, would have been equivalent to finding a needle on a haystack!

Line 150: (Larval development on different substrates). It starts by saying: “Eggs were distributed … My questions are:

(1) Where did the eggs come from? Were they from the general colony or from the three females confined and subjected to different substrates? Please, specify it. I doubt, only three females would be able to generate 100 larvae, especially for the cashew nut tree litter substrate. If the eggs were originated from the colony, I would add: Eggs randomly chosen from our colony were distributed….
(2) Also, add information for the 500ml pots used (Fisher Scientific, cat#, size?).

Line 154: Kept inside of a plastic container….

Were the containers sealed-tight type? Please, specify it.

Line 157: all materials were….

Line 160: It reads: “last adult had from the pupa” It is missing the word emerged. “last adult had emerged from”….

Line 160: I would incorporate the word staggered in this paragraph, to justify why 2 months were necessary to evaluate adult survival from beginning to end and explain to the readers that sand fly larval stages (or instars) don’t change in strict synchronism.

Line 161: Please change hatched to emerged since, adult flies emerge from pupae; while larvae hatch from eggs. Also, check entire manuscript for consistency in usage of both terms.

Line 161: Please, describe brand and size of plastic pots/vials. Were they the same size of the ones used previously for the confinement of three females per substrate?

I would change it by reaffirming: “following strictly the same standard insectary conditions as described above”; since changes in temperature, humidity and photoperiod have drastic impacts on sand fly longevity.

Line 166: The same issue between the usage of hatched vs. emerged words. Knowing that for infections we use adult flies, I would recommend its change to *L. longipalpis* emerged from chicken coops….

Line 221: “The results confirm”…. This should be moved to the conclusion.

Line 223: “*L.longipalpis”* space missing in between *L.* and *longipalpis*. Please, correct it in the entire manuscript.

Line 224: Please, use the word reared instead of raised.

Line 234: Despite of differences in the time needed for larvae to develop according to each specific substrate, why did the authors no try to correlate it with morphology and morphometry of larval stages? Did the morphology and morphometry of larvae, particularly of cashew nut tree substrate, present any particular change when compared to others? Were the adults emerging from this substrate smaller in size, potentially less capable of transmitting Leishmania, than the ones from other substrates? How does the difference in development of larvae compared with time, would correlate to the epidemiology in the field? Would it be possible for us to envision a constant and staggered emergence of flies depending on the substrate of their breeding sites within the same habitat? Would it make the efforts to control Leishmaniasis way more challenging?

Line 252: When we compared, it would read way better than “when we correlated”…. Too repetitive the word “correlated” in the same sentence.

Line 254: change the word detected to observed.

Line 281: (Leishmania infantum infection). Please, change chicken shelter experimental group to chicken coop substrate. Despite of not observing differences in infectivity between flies emerging from chicken coop versus colony substrates, why didn’t the authors:

(1) Attempt to transmit the parasite via the bite of flies?
(2) Attempt to refeed the already infected flies on uninfected blood to investigate the presence of retroleptomonad promastigote development* and possible microbiota changes between different treated groups post 15 days of infection?
(3) Attempt to compare the cashew tree litter substrate to the other two groups? Although justified in the results, of its difficulty as a substrate for rearing flies, I believe, it would have revealed interesting differences in the fly microbiota composition!

Line 310: It would read better: composition of midgut microbiome regardless of lifecycle stage… instead of “that does not take into account lifecycle stage”

Line 320: Remove: “that were reared” and just write reared as larvae on different substrates.

Line 353: shannon index (Capital S for Shannon).

Line 399: Change to “were reared” instead of were cultured.

Line 382-until the end (Discussion):

In my view, the manuscript lacks a better ending or a “grand finale” highlighting the importance of the magnificent, labor intensive and time-consuming research done on the topic. The authors should have left the door open for future experiments by discussing, for example: Why not testing the cashew nut tree litter, as a potential biological control for sand flies, since larvae did poorly on this substrate? What does it make so unique? The cashew gum is said to be an excellent insect repellent and the authors point out its toxicity.

From a sand fly rearing standpoint: The sand fly larval food can either make or break a colony. Would it be feasible, cost efficient and safe to produce the chicken coop larval food in large scale to mass rearing of sand fly colonies and better mimic artificial sand fly infections in the lab? Would it be time to review and alter the one size fits all larval food proposed by Modi and Tesh in 1983? Had the colony flies, the foundation for testing all substrates, been fed on chickens (commonly found in endemic areas) instead of mice, would it have changed the outcome of the results? Had the pots containing the different substrates been prime impregnated with FRASS as described by Wasserber and Rowton, would that alone have drastically changed the outcome of the experiments too? If the number of females placed on each substrate for egg laying, had been increased and males added, would the changes have made a significant difference to the outcome of experiments?

Among the genera of bacteria described, do the authors know if any of them could be useful for a potential biological control in endemic areas of VL, not taking into account, the already in use for mosquito control, Wolbachia? Also, why not to investigate if bacteria could enhance sand fly artificial infectivity or parasite recovery (for flies who infect poorly) in the laboratory? I praise the authors for mentioning the potential for exploring, in future studies, the “harmful effects of *Methylobacterium* in the flies or inmate chemicals properties of cashew nut leaves”.

From a sand fly population genetics standpoint, since the *L. longipalpis* complex occupies long distances with different habitats, including caves in Brazil, would it worth evaluating all putative breeding substrates from highly endemic areas in correlation with morphological, morphometrical, genetical and pheromone-chemical differences already published?

I hope, I have contributed to this manuscript with my modest comments and suggestions.

Please, view them as contributions, not as ways to diminish the quality of the work presented here, to the exciting and sometimes not well funded basic research done on sand flies, particularly regarding to their putative breeding sites in the field and rearing-infecting these pesky insects in the laboratory.

Sincerely Yours,

Referee 2

References:

[G B Modi](https://pubmed.ncbi.nlm.nih.gov/?term=Modi+GB&cauthor_id=6644754), [R B Tesh](https://pubmed.ncbi.nlm.nih.gov/?term=Tesh+RB&cauthor_id=6644754). A simple technique for mass rearing *Lutzomyia longipalpis* and *Phlebotomus papatasi* (Diptera: Psychodidae) in the laboratory.

J Med Entomol. 1983 Oct 5;20(5):568-9.

Gideon Wasserberg and Edgar D. Rowton.

Sub-additive effect of conspecific eggs and frass on oviposition rate of *Lutzomyia longipalpis* and *Phlebotomus papatasi*. J Vector Ecol. 2011 Mar; 36 Suppl 1: S138-43.

[Tiago D Serafim](https://pubmed.ncbi.nlm.nih.gov/?term=Serafim+TD&cauthor_id=29556108) , [Iliano V Coutinho-Abreu](https://pubmed.ncbi.nlm.nih.gov/?term=Coutinho-Abreu+IV&cauthor_id=29556108) , [Fabiano Oliveira](https://pubmed.ncbi.nlm.nih.gov/?term=Oliveira+F&cauthor_id=29556108), [Claudio Meneses](https://pubmed.ncbi.nlm.nih.gov/?term=Meneses+C&cauthor_id=29556108) , [Shaden Kamhawi](https://pubmed.ncbi.nlm.nih.gov/?term=Kamhawi+S&cauthor_id=29556108) , [Jesus G Valenzuela](https://pubmed.ncbi.nlm.nih.gov/?term=Valenzuela+JG&cauthor_id=29556108). Sequential blood meals promote Leishmania replication and reverse metacyclogenesis augmenting vector infectivity. Nat Microbiol. 2018 May;3(5):548-555.
