# Supplementary material for: Effects of larval rearing substrates on some life-table parameters of Lutzomyia longipalpis sand flies
Source: PLoS Negl Trop Dis. 2021 Jan 21;15(1):e0009034. doi: 10.1371/journal.pntd.0009034 (PMC7870073; doi:10.1371/journal.pntd.0009034)
Supplement: Attachment — Submitted filename: Letter with responses.docx [file pntd.0009034.s005.docx]

6^th^ November 2020

Dear Editors and Referees,

In behalf of all authors, I would like to thank you for all suggestions and questions pointed during our previous submission. All questions contributed to the improvement of manuscript and raised new discussions for further projects. Please find below all responses in red color.

Yours sincerely,

Kelsilandia Aguiar Martins

Royal Veterinary College, University of London-UK

kaguiarmartins@rvc.ac.uk

**Responses**

**EDITORIAL**
In addition to the reviewers' comments, I would suggest to modify the graphs in Fig. 1A, 3A, 6C-F, 7 to overlay the graphical representations with the individual data points.

The correction was performed in the new version of manuscript

**REVIEWER #1:** The study is based on sufficient data amount and the study design is appropriate, but the following issues should be completed/added:

Page 8: the description of the method of determination of metacyclic stages in sand flies is missing.
Identification of the microbiota was done from homogenised guts of larvae and adults reared on different substrates. However, the microbiota pattern in the field substrates was not analysed.  In my opinion, this information is substantial and should be provided as it allows comparison of the relative representation of the bacterial genera in the food and sand fly guts (particularly interesting for the representation of Methylobacterium sp. discussed on Page 22)

1- The missing information about infection with *Leishmania* was included with written information and the inclusion of photos of females infected from each group.
2- The microbiota of substrate samples was also sequenced. Samples were diluted in sterile NaCl same as used with dissected guts. Unfortunately, the number of ``*reads``* sequenced in samples from the colony, cashew and chicken coop were too low, even when pooled. The quality of this data makes impossible to distinguish between an actual absence or presence of a specific taxon; or the possibility that the taxon was in the substrate but was not sequenced due to low read counts.

All L1-larvae used here were from disinfected eggs and reared in sterilised material. Besides, all larvae had the same origin, age and equal treatments, but fed with a different source of larval food. Considering such conditions, and the known influence of larval food in the gut microbiota of sand flies larvae, we believe that significant differences of taxa observed looking beta diversity's PCoA analysis in larvae (Figure 6A) are strongly related to substrates offered. These differences of microbiota related to substrates used are reduced when insects became adults (Figure 6B), but this microbiota profile is expected considering physiological changes during pupation. This limitation of a direct association between the microbiota of substrates and sand flies was added to discussion.

**REVIEWER #1:** The results are clearly presented and the figures are of sufficient quality. However, some details on Leishmania infection are missing:
Page 13, lines 281-290 and Fig. 3. There is no information on the colonisation of the stomodeal valve, which is the prerequisite for the successful transmission to the host.  Could authors complete the location of infections either in the form of the figure or just in the text? Presence of metacyclic forms is not specified sufficiently - please provide the relative representation of these forms in both groups.

More detailed information was included across the manuscript about the infection. In addition, photos from both groups showing this region of dissected gut on day 9 was included.

**REVIEWER #3:** - The legend in Figure 3 is swapped: A) represents the parasite load and B) represents the proportion of infected females
- There is no legend for S1 Figure1

The correction was performed in the new version of manuscript

**REVIEWER #3**: In the introduction the authors mention that sand fly breeding sites could be an alternative target for control of leishmaniasis, however in the discussion I expected that the authors would discuss this topic further. Since this research demonstrates that chicken-coop substrate contribute to the development of L. longipalpis and probably to the transmission of L. infantum to dogs and humans, I would suggest to add a few lines about control of these breeding sites in the elimination of Leishmania.

# This subject was more clearly explored in the new version of the manuscript, within topic “Infections with *L. infantum* and epidemiology.’

# REVIEWER #1: Abstract: As no significant differences were found between the experimental group and the control group in susceptibility to Leishmania, the expression about high susceptibility of the experimental group (line 36) is misleading and should be changed.  Better to use the same wording as in the Results (page 13) or Discussion (page 20).

Indeed, no statistical differences were found between both groups, however, it does not disprove the fact that females from chicken coops showed a great proportion of infection and parasite load, same as found in females from colony.

We can find different infections efficiency in sand flies due to *Leishmania* strains, sand flies populations or other biological reasons. If our results show something contrasting, for example, 30% of infection in both groups, certainly would be misleading to say that any group has a good susceptibility. The same misleading would be if results showed something like 40% of infection in chicken group against 20% from colony. Even with a higher proportion of infection than colony group, it still would be a poor infection in chicken coop, different than is usually seen in experimental infections of *Leishmania* *infantum* (<70%).

The terms "leishmaniasis" and "visceral leishmaniasis" should be written consistently with lower-case letters throughout the text (please correct on lines 27, 31, 45, 49, 109-110)

The correction was performed in the new version of manuscript

Page 4, line 75:  in addition to the reference No. 7, also a more recent publication(s) on the development of L. infantum in L. longipalpis and the transmission by the bite should be added (Secundino et al. 2012; Parasites & Vectors 2012, 5:20, Freitas et al. 2012, Am J Trop Med Hyg 2012, 86:4)

The correction was performed in the new version of manuscript

Page 4, line 88: Consider replacing the reference No. 14 with another one, where life span is given for various sand fly species, including L. longipalpis (Volf and Volfova, 2011. J Vector Ecol). Then, you may also discuss if developmental times in your experiments are different from those described in this paper.

The correction was performed in the new version of manuscript

Page 5, lines 98-99: the references 21 and 22 do not contain single evidence about the effect of microbiota on sand fly susceptibility to Leishmania infection. Please correct the sentence or use other references like [53] Kelly et al. 2017, Sant'Anna et al.  (2014) Parasites & Vectors 7:329.

The correction was performed in the new version of manuscript

Page 8. Line 172 – the reference No. 27 does not relate to the method of sand fly infections - should be replaced with a more relevant one.

The correction was performed in the new version of manuscript

Page 16 line 373 – I suppose the word "larval" is a typing error as the whole chapter refers to the adult microbiome.

The correction was performed in the new version of manuscript

In the References section, there are many errors in the use of italics and capitals which must be corrected.
The correction was performed in the new version of manuscript

**REVIEWER #3:** - line 160 "... were carefully monitored for two months after the last adult had from the pupa." There is a missing verb in this sentence.
- line 499 "In females of L. longipalpis...." replace by "Females of L. longipalpis..."

The correction was performed in the new version of manuscript


**REVIEWER #1:** The study evaluates the influence of the larval food, associated with different breeding sites, on the development, microbiome and susceptibility of L. longipalpis to Leishmania. Results of the oviposition assay confirm a well-known fact that sand fly females prefer breeding sites with organic matter rich in nutrition for larvae.  Substrates used for larval rearing differed in many parameters; therefore, it is not surprising that these food types influenced the length of the larval development. On the other hand, the life span of pupae and adults did not differ significantly. The comparison of susceptibility to Leishmania did not reveal any differences between the control group and the group reared on the soil from the chicken shelter. This result is also expectable, as larvae reared on this breeding material developed best from all the tested groups. Evaluation of the susceptibility of females reared on the poor substrate represented here by the cashew litter
would be more interesting, but, as written in the Discussion, the numbers of females emerging on this material were too low to allow the experiment. The analysis of the microbiome showed distinct profiles in both larval stages and adults. However, the results might be better understandable and discussible if authors also provided the analysis of microbiota occurring in the substrates offered to larvae.
Generally, the manuscript is well written but several methodological issues needs to be completed before accepting for publication: identification of microbiota in field substrates, location of Leishmania infection and representation of metacyclic forms in the sand fly midgut. The novelty and significance of findings are limited and data are interesting for a limited number of medical entomology experts only.  Authors may consider transferring the manuscript to more specialised entomological journal (e.g. Med. Vet Entomol.  or J. Med. Entomol)

Indeed, it is well-know the preference of this insect for organic matter. However, one of the novelties of our work relies on the fact that females prefer certain substrates in nature as the breeding site that are not exclusively related to the proportion of organic matter or other nutrients.

Results found here support the idea that other factors as the microbiome or some toxic components found in natural breeding sites can potentially play an important role to *L.longipalpis* biology and perhaps selection of the breeding sites. All these aspects can imply in the epidemiology of the disease considering, for example, the efficient development of the vector and good susceptibility for *Leishmania* in sand flies reared in chicken coops. Therefore, findings in this work are not restricted to lab-based applications. Further investigations focusing on the potential of specific breeding sites to control of the disease can be a strategy explored in future. It is not only for researchers with scientific interest but also for the public politics of control of the disease. In countries like Brazil and others, the management of VL is focused mainly on the host in urban areas (dogs), and one of the reasons is the hard control of unknow breeding sites of this vector.

All non-clear methodological questions pointed here were replied either with new supporting information or with the text included in the manuscript.

**REVIEWER #2:**

I would like to advise the authors to give the paper to a native English speaker for assessment of proper English usage. Here in the US, there are several English teachers, native speakers, who could do it for a small fee. I was able to catch a few mistakes here and there throughout the text.

The new version of manuscript was submitted to professional proofreading considering the American English.

Line 50: Ecological niches versus habitats. What was the intention behind of the sentence "Insects were reared on substrates from different ecological niches"….? Was it a way of emphasising the position of *Lutzomyia longipalpis* within an ecosystem, describing both the range of conditions necessary for insect persistence and its ecological role or merely to inform the putative different substrates where the flies reside?

The intention was to inform about habitats, the word ``niche`` was replaced.

Line 66: Remove: "that had fed on"

Add: "who feed on infected hosts"….

The correction was performed in the new version of manuscript

Line 67-70: It would worth mentioning the mucocutaneous form of the disease; because of its uniqueness and close correlation with some Leishmania species.

The suggestion was included in the new version of manuscript

Line 76: I praise the authors for explicitly recognising *Lutzomyia longipalpis* as a complex of species, well overdue by the sand fly community!

We appreciate the acknowledgment of reviewer.

Line 76: Remove: "where sand flies were feeding on…" Add: where sand flies used to feed exclusively on wild animals and plants; however, with the …..

The correction was performed in the new version of manuscript

Line 82: comprises of residual spraying…..

The correction was performed in the new version of manuscript

Line 86: Add: the word humus after "decaying organic matter or humus.

The correction was performed in the new version of manuscript

Line 95: Suggestion: Although, the term "microbiota" of sand flies was employed correctly, I would review the entire manuscript for consistency; always having in mind the differences between "microbiome" and "microbiota".

The suggestion was considered in the new version of manuscript, and the use of terms followed the vocabulary proposed by Marchesi (2015). (Please find full reference below)

Marchesi JR, Ravel J. The vocabulary of microbiome research: a proposal. *Microbiome*. 2015;3:31. Published 2015 Jul 30. doi:10.1186/s40168-015-0094-5

Line 106 (Study Sites): I would include a brief description of the flora of Jaua and Jacuipe, since substrates from different tree species collected in both areas were analysed in this study. Is one of these tree substrates unique to one of the areas? Is the "coastal strip", a remaining patch of the extensively deforested Atlantic Forest? Please, specify it.

Mango and Orange are exotic plants introduced in Brazil during Portuguese colonization. Only Cashew tree is native from this northeast region of Brazil. Remains of native vegetation are found mainly surrounding the city, where human occupation is still not so intense. It is common to see the replacement of native flora for fruit trees in the peridomicile of such occupied areas in which population can have edible fruits, like mango or orange trees. Probably it explains why we still can find native trees like cashew in the peridomiciles of the region. The environmental characteristics of ``coastal strip", were described in the work of Mota (2018) performed for our group in this same region and cited in the study site.

Line 114 (Insects): Since the foundation of all experiments relied heavily on colonised flies, with either eggs or adults, it is imperative to specify the following:

(1) How many generations the *L. longipalpis* colony, used for all experiments, has been kept in the lab? When was it stablished?

The colony was established by authors of this paper in March 2016, exactly for this project and other further in the lab. All adults that originated the colony were collected in this same region of Camacari. In fact, some of substrates were collected in the same peridomiciles where primary insects that populated the colony were collected.

From the F7 generation we started some trials of each experiment. However, results presented in this manuscript were performed using insects between F9-F26 generation.

(2) How big is the colony?

The graphic bellow summarises our colonisation of *L.longipalpis*. We divided the colonisation in two steps. The first phase, tracking individuals and the second, tracking the number of pots since the tracking became laborious with the growing numbers.

Despite some small issues (common in sand flies colonies), we stabilized the colony with a great success. After stabilized, the density of insects was at least four full cages per week as shown in the picture bellow (Approximately 20.000 insects per week).

Figure- Weekly Production of *L.longipalpis* colony-Fiocruz/BA

(3) What is the yearly production of the colony used for the experiments? Is it mass reared?

Considering this continuous density per week, we estimate almost 1 million per year.

(4) If it is a closed and old colony, how inbreeding and selective pressures by daily rearing methods could impact the outcome of the experiments? If the experiments were done with F1 progeny straight from the field, I assume the authors have access to field collected flies, would it have changed the outcome of the experiments? Please, add some comments on this regard to the discussion section too.

Females were inserted in the colony continually from field. However, the colony was closed since the F9 generation. Results presented here were performed only after this point.

(5) What was the brand of rabbit feed used for maintaining the colony? What was its composition (examples: reach in what nutrients?, specially made or commercially available formula?).

The ration brand was ``Coelhos Trata Mais``. It was based mainly in Wheatbran/Soybean and supplemented with some nutrients. This is the full description of the commercial formula:

Wheat bran, Soybean, Ground whole maise, milled wheat, corn gluten bran 21, ground rice husk, limestone, sodium chloride (common salt), vitamin A / D3, vitamin A, vitamin E, vitamin B1, vitamin B2, folic acid, niacin, calcium pantothenate, calcium iodate, copper sulphate, cobalt sulphate, iron sulphate, manganese sulphate, zinc sulphate, antioxidant additive.

Whole Protein (minimum) ... 120 g / kg

Mineral Matter (maximum) ... 70 g / kg

Fibrous Matter (maximum) ... 100 g / kg

FDA Acid Detergent Fiber (maximum) ... 50 g / kg

Calcium (minimum) ... 10 g / kg

Calcium (maximum) ... 20 g / kg

Phosphorus (minimum) ... 6,000 mg / kg

Sodium (minimum) ... 1,200 mg / kg

Chlorine (minimum) ... 1,800 mg / kg

Humidity (maximum) ... 100 g / kg

(6) What was the source of rabbit feces? Were the rabbits maintained at Fiocruz or bought from outside vendors? Did they receive any antibiotic treatment that might impair the development and microbiome change of larval stages? What was the diet given to the rabbits? Were they kept on an organic diet?

The institute where colony was placed had no license to breed rabbits, then faeces were obtained from home breeding (The author). Initially faeces were bought from a commercial breeder. However, we could not really warranty many of these questions, and the production was not continuous. By using faeces of my own pet it was possible to control closely many of these aspects. The ration offered was the same used in the colony and supplemented with vegetables and fruits.

I would add that larval food was made according to Modi and Tesh, 1983* protocol with a few modifications.

The correction was performed in the new version of manuscript

*References provided at the end of this review.

Lines 127-132 (Substrates): I am really puzzled by this method. How did the authors manage to control fungi growth in larval jars treated with different substrates and applied directly from the field, since the traditional sand fly food preparation method, originally proposed by Young et.al (1981) and latter on simplified by Modi and Tesh in 1983, requires larval food to decompose or "age" for a period of at least 4-6 weeks in a highly humid environment in order for fungi to grow freely, preventing their appearance and trapping of larvae latter on during the sand fly rearing?

"Material was dried, crushed, sieved and stored"…

What was the method used for drying the different substrates? Was it submitted to high temperatures or just air dried? Please specify it, because high temperatures tend to deplete nutrients altering larvae microbiome, this is why larval food shouldn't be autoclaved, particularly when using flies for artificial infection latter on.

1-After collecting from field, each substrate was dried, crushed, and sieved inside a biological hood previously exposed with UV light, then it was frozen in sterile vials of 2ml. These vials were taken from the freezer and placed on pots during each feeding procedure.

These storing conditions could affect the density of fungus. But it is the only option to preserve characteristics of substrates; otherwise, nutritional and microbiota parameters would be completely modified over the natural decomposition at room temperature.

-Indeed, L1 stage can be trapped with large amount of fungi, however, in my experience it happens only when L1 larvae cannot eat the food before a large formation of fungi (because a great amount of food or due to large pierces that would take more time to be consumed). It was the main reason why substrates were crushed and sieved.

Other larvae stages of sand flies in general find in fungi a source of nutrition and only some specify genera are harmful. Some fungi were spotted on pots and larvae fed from them, but we do not have any evidence suggesting a particular effect on profiles found in *L.longipalpis .*

Lines 137-149 (Oviposition Assays): Comment: According to my experience, it would have made a huge difference, if authors had prime impregnated the pots overnight with "FRASS" solution of each individual substrate prior to adding the flies; instead of using the substrates directly, which in my view were highly susceptible to developing mold in the high moisture and temperature conditions.

1-The frass probably would increase the number of eggs; however, our focus was not to find sources of improvement for oviposition. The specific aim here was to study the attractiveness of substrates collected on the field for the oviposition of females. We were trying to explore if those substrates tested could affect the attraction of females differently.

2-Females used to test oviposition were inserted into each pot when they were in the optimal period to start the oviposition (72 hours post blood meal). Therefore, all females were attracted and ready to place eggs when substrates still were with all characteristics intact and without external interferences.

Please refer to the work of Wasserberg and Rowton (2011)*. This is done regularly to rapidly increase colony flies, regardless of species, with great improvement in number of eggs oviposited by each female fly!!!

We agree, we have used such method during the establishment of our colony, using old pots.

Line 139: Please specify the pot or vial used (brand, catalog number, size, etc.). Were they Falcon tubes? Self-standing tubes? 3-dram vials?

Polyethylene pots/acrylic pots (disposable plastic pots) were used during all experiments. We decided not to re-use pots from none of these experiments for logistic reasons and some concerns of contamination for the colony.

Line 140: Not all plasters are created equal! Please specify the brand used, if dental or plaster for dry wall construction (still releasing chemicals that could impact egg laying).

The brand was included on methods. The plaster for wall construction was used since the beginning of colony, eggs were on average expected per females (30-50eggs). Since no differences were observed we continued using the same type. The plaster used is the most basic type without other components that sometimes can be included (like glue).

Also, old pots (pots that have passed through the colony several times) tend to be more productive in terms of egg laying, since they retain better moisture and "odors" released by female flies during ovipositing and humus impregnating, refer to Wasserberg and Rowton work, than freshly made ones. Did the authors consider this critical piece of information when designing the experiments for this study?

Such method was used only during the initial establishment of our colony, because in that scenario the enhancement of oviposition was something we tried to achieve. However, like also mentioned before, a better attraction of females for oviposition was not the aim of our experiments.

Line 144: How did the authors determine the optimal number of females to be confined in each pot? Did they take into account that aggregation plays a major role in sand fly ovipositing? Why male flies were not added to each pot? Some laboratory observations suggest that male flies help during ovipositing and blood meal acquiring of female flies. We have observed a significant improvement in sand fly artificial feeding when males (5-10% of total) are added to containers with females. Did the authors take into account the size of the pots and the fact that *L. longipalpis* oviposits better when confined in tiny spaces? How was the density of flies per area (cm^2^) determined? Empirically?

1-Some trials were performed using colony food to find the minimal number of females per experiment. When we used 1-2 females just a small number of pots contained eggs. It is perhaps related to the pot size. The aggregation behavior was considered, but we believe this presence did not invalidate our results considering the experimental design and results:

1-Each group of females were exposed to a specific type of substrate 12x considering the triplicate of dual tests. For instance:

2-Eggs found per each trial rarely were concentrated into a single pot, the contrast among substrates of each trial was observed by the proportion of eggs.

3- Since we were exclusively trying to find possible differences in the attractiveness for oviposition linked to substrates from field, males were not included to enhance the oviposition of *L.longipalpis.* However, all females were exposed with males since their emergence up the moment they were transferred to experimental pots.

Line 145: Under standard laboratory conditions of 65%RH - 27^0^C, used for general rearing, *L. longipalpis* flies require an average of 7 to 10 days to fully oviposit their eggs. This can be accelerated or retarded by changing temperature, humidity and photoperiod conditions. Questions:

(1) Why did the authors decide to interrupt the egg laying after 72 hours?
(2) What was the reasoning behind it? Did the adult flies simply die after 72 hours or was it cut short because mold from raw substrates started building up, trapping the flies? This is why I continue to insist that FRASS impregnation would have helped a lot!
(3) If they had left the flies for a longer period, would they have oviposited more eggs per substrate?
(4) Did the authors consider the possibility of autogeny, already described in long term colonised flies, when designing the experiments?

(1,2,3,) Females were placed on pots 72h after the blood feeding, exactly when they start to lay eggs and extra 3 days to allow the oviposition. During our initial trials using 20 females placed individually on pots we found that majority of females placed their eggs during this period of 6 days post blood ingestion(n=16). The speed of oviposition can change with many aspects and a ``window `` for the oviposition like from this work was seen previously **(Moraes, 2018)**. Consistently, **Magnarelli (1984**) showed that *L. longipalpis* contained 69% of mature oocytes on 6th day post blood ingestion at similar temperatures. Then , we believe that just a small number of females probably would lay some eggs after the period evaluated during our experiments. More importantly, we found that leaving to check eggs after this period would result in finding also fungi on pots that could disturb an accurate counting of eggs.

The fact that we did not wait to check eggs later did not compromise the main aim of experiment considering this context. Moreover, the same number of females was used among all groups, eggs number were evaluated after the same period and triplicates were performed for each dual of substrates tested. (Please find the references cited bellow)

(4) Considering that our colony was recently established and the main aims of this experiment, autogeny was not a factor considered.

Moraes CS, Aguiar-Martins K, Costa SG, Bates PA, Dillon RJ, Genta FA. Second Blood Meal by Female *Lutzomyia longipalpis*: Enhancement by Oviposition and Its Effects on Digestion, Longevity, and *Leishmania* Infection. *Biomed Res Int*. 2018;2018:2472508. Published 2018 Mar 25. doi:10.1155/2018/2472508

Magnarelli, LA; Modi, GB; Tesh, RB. Follicular development and parity in phlebotomine sand flies (Diptera: Psychodidae). J. Med. Entomol. Vol. 21, 681-689,1984.

Line 148: I am highly puzzled by this method too! How were the small microscopic sand fly eggs of just three females scooped out from pots and counted, while still mixed with the different substrates? This alone, would have been equivalent to finding a needle on a haystack!

Perhaps the perception of food volume usually used to feed larvae on colony pots could be a factor of confusion. A small volume of substrate was spread across the bottom of each small pot and the plaster was still visible (Please see the photo below). Unfortunately, I do not have a good photo showing clearly eggs in these pots, but this used can offer some idea of proportion. We did not have any problem to spot the shiny shape of dark eggs in these conditions. I believe that other factor that contributed strongly to accurate counting was the fact that substrates were sifted; only small pierces of food were seen on pots. Lastly, all pots were observed before fungi could also grow and hide eggs placed.

Line 150: (Larval development on different substrates). It starts by saying: "Eggs were distributed … My questions are:

(1) Where did the eggs come from? Were they from the general colony or from the three females confined and subjected to different substrates? Please, specify it. I doubt, only three females would be able to generate 100 larvae, especially for the cashew nut tree litter substrate. If the eggs were originated from the colony, I would add: Eggs randomly chosen from our colony were distributed….

(1) Eggs were collected from general colony. It is now clarified in the text.

(2) Also, add information for the 500ml pots used (Fisher Scientific, cat#, size?).
(2) The information was included in the new version of manuscript

Line 154: Kept inside of a plastic container….

Were the containers sealed-tight type? Please, specify it.

The information was included in the new version of manuscript

Line 157: all materials were….

The sentence was corrected in the new version of manuscript

Line 160: It reads: "last adult had from the pupa" It is missing the word emerged. "last adult had emerged from"….

The sentence was corrected in the new version of manuscript

Line 160: I would incorporate the word staggered in this paragraph, to justify why 2 months were necessary to evaluate adult survival from beginning to end and explain to the readers that sand fly larval stages (or instars) don't change in strict synchronism.

The information was included in the new version of manuscript

Line 161: Please change hatched to emerged since, adult flies emerge from pupae; while larvae hatch from eggs. Also, check entire manuscript for consistency in usage of both terms.

This information was corrected in the new version of manuscript

Line 161: Please, describe brand and size of plastic pots/vials. Were they the same size of the ones used previously for the confinement of three females per substrate?

The information was included in the new version of manuscript

I would change it by reaffirming: "following strictly the same standard insectary conditions as described above"; since changes in temperature, humidity and photoperiod have drastic impacts on sand fly longevity.

The information was included in the new version of manuscript

Line 166: The same issue between the usage of hatched vs. emerged words. Knowing that for infections we use adult flies, I would recommend its change to *L. longipalpis* emerged from chicken coops….

This information was corrected in the new version of manuscript

Line 221: "The results confirm"…. This should be moved to the conclusion.

Line 223: "*L.longipalpis"* space missing in between *L.* and *longipalpis*. Please, correct it in the entire manuscript.

This information was corrected in the new version of manuscript

Line 224: Please, use the word reared instead of raised.

This information was corrected in the new version of manuscript

Line 234: Despite of differences in the time needed for larvae to develop according to each specific substrate, why did the authors no try to correlate it with morphology and morphometry of larval stages? Did the morphology and morphometry of larvae, particularly of cashew nut tree substrate, present any particular change when compared to others? Were the adults emerging from this substrate smaller in size, potentially less capable of transmitting Leishmania, than the ones from other substrates?

Characteristics like differences of morphology observed in adults from different groups were mainly descriptive since it was not initially our aim. However, due to visible contrast between groups like chicken and cashew it was a piece of information included. Larvae differences among these groups did not attract our attention during observations (not a visible distinction in size or colour). However, a more detailed investigation certainly would be interesting to explore.

How does the difference in development of larvae compared with time, would correlate to the epidemiology in the field? Would it be possible for us to envision a constant and staggered emergence of flies depending on the substrate of their breeding sites within the same habitat? Would it make the efforts to control Leishmaniasis way more challenging?

Considering the example of contrasting performance in cashew LL and chicken coops , as an example, we can have a perspective of the effect in field. Habitats like chicken coops offer the chance of a larger population of *L.longipalpis* in endemic areas in a shorter period of time when compared with others like Cashew -LL. Considering that females prefer to lay their eggs in places that favor their offspring I believe that is likely that they would try to find a better place for oviposition than Cashew -LL. Therefore, our results suggest that in fact we can narrow breeding sites choices of *L.longiplapis* since they do not lay randomly their eggs. In addition, we found that habitats like chicken coops can contribute with the manutention of sand flies population in endemic areas not only as a source of blood. The presence of habitats like chicken coop certainly contribute to density of *L.longipalpis* considering the good source of larval food and females blood source. The fact that it also work was a great breeding site suggested that it could be strategic place for the control of the vector.

Results found in chicken coops are more meaningful to epidemiology of LV when we consider also the great susceptibility of insect raised in this substrate to *L.infantum.* Our work clarifies that the role hole of chiken coops are not restricted to blood source.

Line 252: When we compared, it would read way better than "when we correlated"…. Too repetitive the word "correlated" in the same sentence.

This information was corrected in the new version of manuscript

Line 254: change the word detected to observed.

This information was corrected in the new version of manuscript

Line 281: (Leishmania infantum infection). Please, change chicken shelter experimental group to chicken coop substrate. Despite of not observing differences in infectivity between flies emerging from chicken coop versus colony substrates, why didn't the authors:

(1) Attempt to transmit the parasite via the bite of flies?
(2) Attempt to refeed the already infected flies on uninfected blood to investigate the presence of retroleptomonad promastigote development* and possible microbiota changes between different treated groups post 15 days of infection?
(3) Attempt to compare the cashew tree litter substrate to the other two groups? Although justified in the results, of its difficulty as a substrate for rearing flies, I believe, it would have revealed interesting differences in the fly microbiota composition!

The transmission and other aspects asked here were not explored due to limitations of time available to finish the project. However, we are applying at moment for a small project grant including such questions.

Line 310: It would read better: composition of midgut microbiome regardless of lifecycle stage… instead of "that does not take into account lifecycle stage"

This information was corrected in the new version of manuscript

Line 320: Remove: "that were reared" and just write reared as larvae on different substrates.

This information was corrected in the new version of manuscript

Line 353: shannon index (Capital S for Shannon).

This information was corrected in the new version of manuscript

Line 399: Change to "were reared" instead of were cultured.

This information was corrected in the new version of manuscript

Line 382-until the end (Discussion):

In my view, the manuscript lacks a better ending or a "grand finale" highlighting the importance of the magnificent, labor intensive and time-consuming research done on the topic. The authors should have left the door open for future experiments by discussing, for example: Why not testing the cashew nut tree litter, as a potential biological control for sand flies, since larvae did poorly on this substrate? What does it make so unique? The cashew gum is said to be an excellent insect repellent and the authors point out its toxicity.

This suggestion was included in the new version of manuscript

From a sand fly rearing standpoint: The sand fly larval food can either make or break a colony. Would it be feasible, cost efficient and safe to produce the chicken coop larval food in large scale to mass rearing of sand fly colonies and better mimic artificial sand fly infections in the lab? Would it be time to review and alter the one size fits all larval food proposed by Modi and Tesh in 1983? Had the colony flies, the foundation for testing all substrates, been fed on chickens (commonly found in endemic areas) instead of mice, would it have changed the outcome of the results? Had the pots containing the different substrates been prime impregnated with FRASS as described by Wasserber and Rowton, would that alone have drastically changed the outcome of the experiments too? If the number of females placed on each substrate for egg laying, had been increased and males added, would the changes have made a significant difference to the outcome of experiments?

1-Considering, the small difference of efficiency between results of colony food and chicken coops, besides the strong smell found in chicken faeces, I do not believe would worth the replacement. Results found here have an epidemiological/biological appeal rather than colony applications, despite the majority of experiments were performed in lab conditions.

2-The blood-feeding source can affect the oviposition rate of sand flies. Santana (2010) showed that *L. longipalpis* fed on chickens or dogs produced significantly more eggs than those fed on human blood, despite the longevity after feeding was not affected or blood digestion. This comparison of oviposition was not explored with hamster, but even considering a lower egg's production using hamsters, our main aim here of comparing substrates attractiveness would be not affected. For experiments looking the development of sand flies we used a fixed number of eggs from colony, then the source of blood would not matter (Please find the citation below).

Sant'Anna, M.R., Nascimento, A., Alexander, B. *et al.* Chicken blood provides a suitable meal for the sand fly *Lutzomyia longipalpis* and does not inhibit *Leishmania* development in the gut. *Parasites Vectors* **3,** 3 (2010). <https://doi.org/10.1186/1756-3305-3-3>

3-We believe the frass could attract more females. However, results found here would be not exclusively related to attraction by substrates, but also probably to the gut microbiota of larvae. The frass improves the attraction for oviposition, however it is not required to females choose where to lay their eggs.

4-Considering that same condition would be offered among all groups, the inclusion of males would not affect the main aim of compare the attractiveness of substrates. However, to include too much females could interfere on specificity of results considering a stronger effect of the`` aggregation effect ``.

Among the genera of bacteria described, do the authors know if any of them could be useful for a potential biological control in endemic areas of VL, not taking into account, the already in use for mosquito control, Wolbachia? Also, why not to investigate if bacteria could enhance sand fly artificial infectivity or parasite recovery (for flies who infect poorly) in the laboratory? I praise the authors for mentioning the potential for exploring, in future studies, the "harmful effects of *Methylobacterium* in the flies or inmate chemicals properties of cashew nut leaves".

1-The genera described here was still not tested against *Leishmania.*

*2-*The question of referee about exploring specific bacteria during sand fly artificial

is certainly interesting, however it was not included in our project considering the time.

From a sand fly population genetics standpoint, since the *L. longipalpis* complex occupies long distances with different habitats, including caves in Brazil, would it worth evaluating all putative breeding substrates from highly endemic areas in correlation with morphological, morphometrical, genetical and pheromone-chemical differences already published?

Our results showed that the food source affected the size and colour of insects. I believe it could be a physiological effect on insects' body due to components present in each type of larval food. I suspect that darker colours are related to components of feces, since insects from chicken and duck coops appeared with darker colour, by other hand all insects fed from leaves showed a lighter brown colour. I would be interested in investigating specifically the correlation of this aspect with source of food.
